# Use of artificial nutrition near the end of life: Results from a French national population‐based study of hospitalized cancer patients

**DOI:** 10.1002/cam4.2731

**Published:** 2019-11-26

**Authors:** Karine Baumstarck, Laurent Boyer, Vanessa Pauly, Veronica Orleans, Anthony Marin, Guillaume Fond, Lucas Morin, Pascal Auquier, Sébastien Salas

**Affiliations:** ^1^ CEReSS—Health Service Research and Quality of Life Center Aix‐Marseille Univ. Marseille France; ^2^ Department of Epidemiology and Health Economics APHM Marseille France; ^3^ Department of Medical Information APHM Marseille France; ^4^ Department of Adult Oncology APHM Marseille France; ^5^ Department of Medical Epidemiology and Biostatistics Karolinska Institutet Stockholm Sweden; ^6^ Inserm CIC 1431 University Hospital of Besançon Besançon France

**Keywords:** artificial nutrition, cancer, end‐of‐life, health services research, registry database

## Abstract

**Background:**

The use of artificial nutrition, defined as a medical treatment that allows a non‐oral mechanical feeding, for cancer patients with limited life expectancy is deemed nonbeneficial. High‐quality evidence about the use of artificial nutrition near the end of life is lacking. This study aimed (a) to quantify the use of artificial nutrition near the end‐of‐life, and (b) to identify the factors associated with the use of artificial nutrition.

**Methods:**

This was a retrospective cohort study of decedents based on data from the French national hospital database. The study population included adult cancer patients who died in hospitals in France between 2013 and 2016 and defined to be in a palliative condition. Use of artificial nutrition during the last 7 days before death was the primary endpoint.

**Results:**

A total of 398 822 patients were included. The median duration of the last hospital stay was 10 (interquartile range, 4‐21) days. The artificial nutrition was used for 11 723 (2.9%) during the last 7 days before death. Being a man, younger, having digestive cancers, metastasis, comorbidities, malnutrition, absence of dementia, and palliative care use were the main factors associated to the use of artificial nutrition.

**Conclusion:**

This study indicates that the use of artificial nutrition near the end of life is in keeping with current clinical guidelines. The identification of factors associated with the use of artificial nutrition, such as cancer localization, presence of comorbidities or specific symptoms, may help to better manage its use.

## Graphical abstract

In France, the artificial nutrition management is rather in accordance with the available clinical guidelines for cancer patients with limited life expectancy.

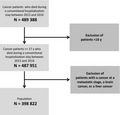

## 1 INTRODUCTION

During the last decades, the care management of advanced cancer patients near the end of life has prioritized symptom management, psychological support, and advanced care planning to preserve quality of life. Cancer care in the context of limited life expectancy is a challenge (clinically and ethically) that physicians have to face in their daily practice.^1^

Several factors have been shown to have no or negative effects on patients' outcomes during this specific time. The use and/or continuation of aggressive care (in particular chemotherapy) close to death is widely deemed nonbeneficial due to a limited effect on overall survival, symptom deterioration, and the high burden of toxicity, leading to impairments (or the absence of improvement) in quality of life.^2^, ^3^, ^4^, ^5^ The early integration of palliative care into standard oncological care seems to be associated with improved patient outcomes in the last days before death.^6^, ^7^ However, the question of whether to maintain artificial nutrition, defined as a medical treatment that allows a non‐oral mechanical feeding either by intravenous or enteral route for a person no longer able to take nutrition by mouth, is still discussed.

While artificial nutrition is a key component of care for cancer patients in general (eg, prevention of weight loss and malnutrition, prevention of side‐effects from anticancer treatments),^8^, ^9^ its use for patients with a shortened life expectancy appears nonbeneficial.^10^, ^11^ In fact, the implementation of artificial nutrition in the terminal and dying phase may be considered a health risk (eg, infectious, respiratory, metabolic disorders^12^), may result in the discomfort of the patient, and may be a source of health costs.

Few studies have reported robust information about the use of artificial nutrition for patients with limited life expectancy. Based on a 2011 review, the frequency of use of artificial nutrition in the last week of life varied between studies, from 3% to 50%.^13^ A recent French study, focusing on patients with metastatic oesophageal/stomach cancer who died between 2010 and 2013, reported that more than 15% of the patients received artificial nutrition in the last week of life,^14^ while the French Society of Clinical Nutrition and Metabolism recommends artificial nutrition only in patients with an expected life‐expectancy greater than 3 months (https://sfncm.org/images/stories/Reco_oncologie_final.pdf; last visit 2018, December). We may hypothesize that patients receiving artificial nutrition differ from other patients in terms of the presence of specific symptoms or malnutrition or needs in the last days of life. A better understanding of the factors associated with the use of artificial nutrition may help clinicians better manage its use and may improve communication with patients, families, and health policy makers. We provide, for the first time, robust information about the use of artificial nutrition during the last days before death from the French national hospital database.

The aims of this study were (a) to quantify the use of artificial nutrition near the end‐of‐life (during the 7 and 31 last days of life^15^) for hospitalized cancer patients, and (b) to identify the factors associated with the use of artificial nutrition.

## 2 METHOD

### 2.1 Design

This was a French population‐level retrospective cohort study based on data extracted from the French national hospital database (Programme de Médicalisation des Systèmes d'Information) including administrative and medical information collected in both public and private hospitals in France. Diagnoses are coded according to the International Classification of Diseases, Tenth Revision (ICD‐10).

### 2.2 Population

The study population included all cancer patients aged 17 years and older who died during a hospital stay between 1 January 2013 and 31 December 2016. Patients were identified using the algorithm developed by the French National Cancer Institute (French, *Institut National du Cancer* [INCa]), the algorithm was specifically designed to identify cancer‐related treatments with routinely collected data (INCa 2013). Patients were included if they were in a palliative care situation according to the definition used in previous works involving the French national hospital database^16^: diagnosed with cancer at a metastatic stage, brain cancer, or liver cancer (regardless of the stage of the disease as these tumors are rarely metastatic and are usually lethal at locoregional stages). Patient care managed in rehabilitation centers and at home could not be included given the nature of the registry. The access to this national database is granted according to the French law and is available for accredited provider.

### 2.3 Outcomes

Two main outcomes were studied: the use of artificial nutrition during: (a) the last 7 days before death (primary outcome) and (b) the last 31 days before death (secondary outcome). The rationale for the last 7 and 31 days before death was based on the Earle's work.^15^

The use of artificial nutrition was defined as the presence of enteral or parenteral nutrition related to acute care during a hospitalization stay, using the French classification of medical acts (https://www.ameli.fr/accueil-de-la-ccam/index.php) (codes are provided in the Appendix). Enteral nutrition includes nutrition provided through either nasogastric tubes or gastrostomy, esophogostomy, or jejunostomy tubes. Parenteral nutrition includes nutrition administered through a peripheral vein or a central line called total parenteral nutrition.

### 2.4 Factors associated with artificial nutrition

The following data were extracted:

- Sociodemographic information: age, gender, and year of death;
- Characteristics of the last hospitalization stay before death: duration and type of hospital (specialized or nonspecialized hospital);
- General clinical information: cancer localization, chronic comorbid condition based on the modified Charlson comorbidity index^17^ (score computed as the number of comorbidities excluding cancer‐related items), presence and severity of malnutrition (five classes: absence, mild, moderate, severe, and undefined severity), presence of a biological/clinical situation that is potentially source a reason for the implementation/maintenance of artificial nutrition (eight items: cachexia, anorexia, metabolic disorders (dysnatremia, dyskalemia, others), mucositis/stomatitis, hepatic disorders (acute and chronic hepatitis, hepatic failures…), digestive symptoms, respiratory symptoms, skin ulcerations), and dementia. All the codes and labels are detailed in the Appendix. The rationale for selecting the specific factors was based on biological or clinical situations potentially source of prescription or non‐prescription of artificial nutrition.
- Care management during the last 7 and 31 days before death: chemotherapy use and palliative care use (Appendix).

### 2.5 Statistical analysis

Descriptive analyses of socio‐demographic information, hospital stay, general clinical information, and care management were presented as frequencies and percentages. The percentages of patients who received artificial nutrition in the last 7 and 31 days before death were calculated. Each outcome was used as a separate dependent variable. Univariate associations between sociodemographic information, hospital stay, general clinical information, and care management and each outcome were performed using univariate logistic regression. Variables relevant to the model were selected based on a threshold *P*‐value (≤.05) in the univariate analysis and included in a multivariate logistic model to predict the odds of receiving artificial nutrition in the last 7 and 31 days before death. Adjusted odds ratios (ORs) with 95% confidence intervals (95% CIs) were calculated. Statistical significance was defined as *P* < .05. The statistical analysis was performed in accordance with the REporting of studies Conducted using Observational Routinely‐collected health Data statement. The statistical analysis was performed with SAS 9.4 (SAS Institute) and the logistic regression used the PROC LOGISTIC model in SAS.

## 3 RESULTS

### 3.1 Population

**Figure 1:**
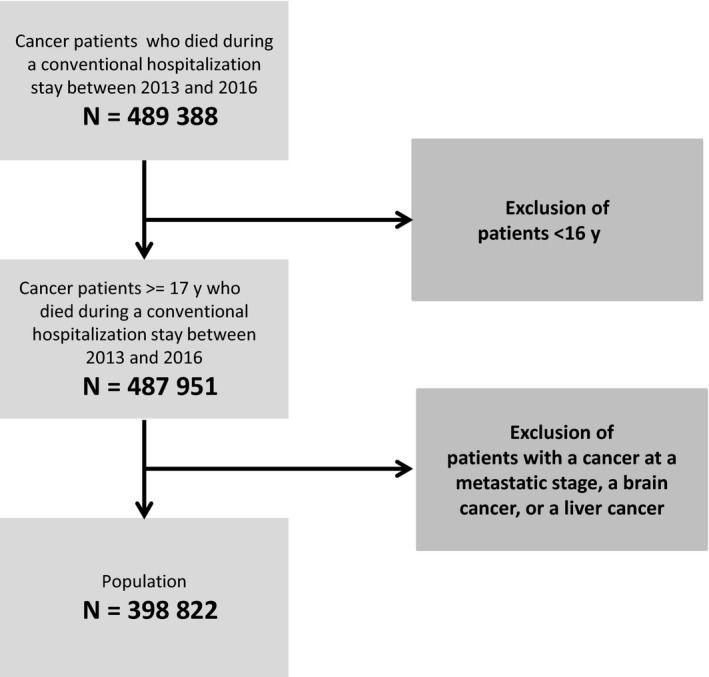
Flow diagram

A total of 398 822 hospitalized cancer patients who died between 1 January 2013 and 31 December 2016 met the inclusion criteria. A flow diagram detailing the selection of cases is shown in Figure 1. The median duration of the last hospitalization was 10 days (interquartile range 4‐21) and the duration was less than 7 days in almost 40% of the patients. The patients were predominantly men (59%), and 30% of them were aged over 80 years at the time of death. Digestive cancers (34%), including pancreatic (7%) and oesophageal (6%) cancers, and lung cancer (24%) were the most frequent cancers among the patients. Sixty percent of the patients presented at least one chronic comorbidity according to the modified Charlson index. One‐fifth of the patients received chemotherapy in the last 31 days before deaths vs 8% in the last 7 days before death. One quarter of the patients were not labeled as receiving palliative care. All the details are shown in Table 1. The use of artificial nutrition during the last 7 days and the last 31 days before death was found in 11 723 (2.9%) patients and 20 429 (5.1%) patients, respectively.

**Table 1:** Characteristics N = 398 822

	N (%)
Sex	
Women	162 506 (40.8)
Men	236 316 (59.3)
Age at death (years)	
<60	77 469 (19.4)
[60;70]	102 760 (25.8)
[70;80]	101 295 (25.4)
≥80	117 298 (29.4)
Year of death	
2013	96 868 (24.3)
2014	98 948 (24.8)
2015	100 383 (25.2)
2016	102 623 (25.7)
Last hospitalization stay duration	
≥7 d	242 071 (60.7)
≥31 d	47 952 (12.0)
Hospital type	
Non specialized^a^	300 347 (75.3)
Specialized^a^	98 475 (24.7)
Cancer localization	
Digestive	134 934 (33.8)
Pancreas	29 660 (7.4)
Esophageal	23 340 (5.9)
Others	81 934 (20.5)
Lung/thoracic	95 068 (23.8)
Genital (male and female)	44 368 (11.1)
Breast	34 193 (8.6)
Hematologic	32 320 (8.1)
Urinary tract	29 099 (7.3)
Head and neck	18 635 (4.7)
Nervous system	11 960 (3.0)
Others^b^	17 289 (4.3)
Unknown primary site	8170 (2.1)
Comorbidities (Charlson)^c^	
0	160 316 (40.2)
1 or 2	141 498 (35.5)
3 or 4	64 112 (16.1)
≥5	32 896 (8.3)
Diabete without complication	72 634 (18.2)
Congestive heart failure	67 122 (16.8)
Chronic pulm. disease	54 515 (13.7)
Renal disease	44 671 (11.2)
Malnutrition	
The last 7 d	119 296 (29.9)
The last 31 d	147 775 (37.1)
Chemotherapy use	
The last 7 d	31 926 (8.0)
The last 31 d	85 580 (21.5)
Palliative care use	
The last 7 d	297 012 (74.5)
The last 31 d	301 424 (75.6)

a Specialized centers include cancer units of an university hospital and units of a cancer hospital, non‐specialized centers include all the other cases.

b Others: skin (2.52%), bone (1.10%), endocrine glands (0.58%), eye (0.14%).

c Charlson modified score (excluding malignancies/metastasis).

### 3.2 Factors associated with artificial nutrition during the last 7 days before death

Artificial nutrition was more often received by men, younger individuals, and patients with longer hospitalization durations. The presence of higher number of comorbidities, presence of malnutrition, and the following clinical/biological items were more often associated with the use of artificial nutrition: cachexia, metabolic disorders, mucositis/stomatitis, hepatic disorders, digestive symptoms, respiratory symptoms, and skin ulceration. Artificial nutrition was used more often for digestive and head‐and‐neck localizations than for other cancers. The presence of metastasis and dementia were less often associated with the use of artificial nutrition. Chemotherapy use during the last 7 days before death was more often associated with the use of artificial nutrition, while the use of palliative care was less often associated with the use of artificial nutrition. The use of artificial nutrition was slightly less frequent in 2015. The multivariate models confirmed these findings, except for the findings regarding chemotherapy use. The univariate analysis results are detailed in Table 2 and the multivariate analysis results are detailed in Table 3.

**Table 2:** Factors associated to artificial nutrition use in the last 7 and 31 d before death (univariate analysis)

	The last 7 d before death			The last 31 d before death		
	N = 11 723 (2.9%)	OR (95% CI)	*P*	N = 20 429 (5.1%)	OR (95% CI)	*P*
	N (%)			N (%)		
Sex						
Women (ref)	3932 (2.4)			7252 (4.5)		
Men	7591 (3.2)	1.34 (1.29‐1.39)	**<.001**	13 177 (5.6)	1.26 (1.23‐1.3)	**<.001**
Age at death (years)						
<60 (ref)	2904 (3.8)			5223 (6.7)		
[60;70]	3691 (3.6)	0.96 (0.91‐1.01)	.079	6495 (6.3)	0.93 (0.90‐0.97)	**<.001**
[70;80]	3153 (3.1)	0.82 (0.78‐0.87)	**<.001**	5486 (5.4)	0.79 (0.76‐0.82)	**<.001**
≥80	1775 (1.5)	0.39 (0.37‐0.42)	**<.001**	3225 (2.8)	0.39 (0.37‐0.41)	**<.001**
Year of death						
2013 (ref)	2869 (3.0)			5019 (5.2)		
2014	2908 (2.9)	0.99 (0.94‐1.05)	.77	5216 (5.3)	1.02 (0.98‐1.06)	.370
2015	2744 (2.7)	0.92 (0.87‐0.97)	**.002**	4977 (5.0)	0.95 (0.92‐0.99)	**.024**
2016	3002 (2.9)	0.99 (0.94‐1.04)	.630	5217 (5.1)	0.98 (0.94‐1.02)	.323
Hospitalization stay duration (days)						
≤7 (ref)	3895 (2.5)			5924 (3.8)		
[8‐31]	6002 (3.1)	1.25 (1.2‐1.3)	**<.001**	11 492 (5.9)	1.60 (1.55‐1.65)	**<.001**
>31	1626 (3.4)	1.38 (1.3‐1.46)	**<.001**	3013 (6.3)	1.71 (1.63‐1.79)	**<.001**
Hospital type						
Non speciality	7330 (2.4)			13 334 (4.4)		
Speciality	4193 (4.3)	1.78 (1.71‐1.85)		7095 (7.2)	1.67 (1.62‐1.72)	**<.001**
Cancer localization						
NDHAN^a^ (ref)	6251 (2.5)			10 587 (4.2)		
Digestive^b^	4230 (3.2)	1.31 (1.26‐1.36)	**<.001**	7735 (5.9)	1.43 (1.39‐1.47)	**<.001**
Head and neck	1042 (6.2)	2.57 (2.4‐2.75)	**<.001**	2107 (12.5)	3.23 (3.08‐3.40)	**<.001**
Metastasis						
(ref: no)	7044 (2.8)	0.87 (0.84‐0.9)	**<.001**	12 658 (4.9)	0.90 (0.87‐0.93)	**<.001**
Comorbidities (Charlson)^c^						
Absence (ref)	3930 (2.5)			7341 (4.6)		
1 or 2	4363 (3.1)	1.27 (1.21‐1.32)	**<.001**	7585 (5.4)	1.18 (1.14‐1.22)	**<.001**
3 or 4	2035 (3.2)	1.3 (1.24‐1.38)	**<.001**	3474 (5.4)	1.19 (1.15‐1.24)	**<.001**
≥5	1195 (3.6)	1.5 (1.4‐1.6)	**<.001**	2029 (6.2)	1.37 (1.3‐1.44)	**<.001**
Malnutrition						
Absence (ref)	6572 (2.4)			9072 (3.6)		
Mild	268 (6.1)	2.68 (2.36‐3.04)	**<.001**	533 (9.2)	2.71 (2.47‐2.97)	**<.001**
Moderate	1343 (3.9)	1.69 (1.60‐1.80)	**<.001**	2930 (6.7)	1.93 (1.84‐2.01)	**<.001**
Severe	3037 (4.1)	1.79 (1.71‐1.87)	**<.001**	7294 (8.1)	2.35 (2.28‐2.42)	**<.001**
Other cases^d^	303 (4.3)	1.85 (1.65‐2.08)	**<.001**	600 (7.2)	2.06 (1.89‐2.25)	**<.001**
Presence of specific situation(s)^e^, ^f^						
No (ref)	3070 (2.1)			3722 (3.3)		
At least one^g^	8453 (3.4)	1.66 (1.59‐1.73)	**<.001**	16 707 (5.9)	1.86 (1.79‐1.93)	**<.001**
Cachexia						
(ref: no)	628 (3.3)	1.14 (1.05‐1.23)	**.002**	1451 (6.6)	1.33 (1.26‐1.41)	**<.001**
Anorexia						
(ref: no)	673 (2.8)	0.95 (0.88‐1.02)	.175	1807 (5.7)	1.12 (1.07‐1.18)	**<.001**
Metabolic disorders						
(ref: no)	4578 (4.5)	1.99 (1.92‐2.07)	**<.001**	9121 (0.07)	1.86 (1.81‐1.91)	**<.001**
Mucositis/stomatitis						
(ref: no)	617 (3.3)	1.16 (1.06‐1.26)	**<.001**	1591 (6.8)	1.39 (1.32‐1.46)	**<.001**
Hepatic disorders						
(ref: no)	1575 (4.3)	1.58 (1.49‐1.66)	**<.001**	2689 (6.6)	1.35 (1.29‐1.41)	**<.001**
Digestive symptoms						
(ref: no)	2711 (3.5)	1.28 (1.22‐1.33)	**<.001**	6529 (6.8)	1.51 (1.46‐1.55)	**<.001**
Respiratory symptoms						
(ref: no)	1604 (3.5)	1.26 (1.2‐1.33)	**<.001**	3459 (6.3)	1.3 (1.25‐1.35)	**<.001**
Skin ulceration						
(ref: no)	1732 (3.2)	1.12 (1.06‐1.18)	**<.001**	3771 (6.2)	1.28 (1.23‐1.32)	**<.001**
Dementia						
(ref: no)	208 (1.5)	0.5 (0.44‐0.58)	**<.001**	446 (2.8)	0.52 (0.47‐0.57)	**<.001**
Chemotherapy use^f^						
(ref: no)	1236 (3.9)	1.4 (1.31‐1.48)	**<.001**	5406 (6.3)	1.34 (1.3‐1.38)	**<.001**
Palliative care use^f^						
(ref: no)	6492 (2.2)	0.43 (0.41‐0.45)	**<.001**	13 563 (4.5)	0.62 (0.6‐0.64)	**<.001**

Note

Bold values: *P*‐values < .05.

Abbreviations: OR (95% CI), odd ratio with the 95% confidence interval; Ref, modality of reference.

a NDHAN: Non‐digestive and non‐head‐and‐neck cancers.

b Digestive cancers: oesophageal, pancreas, and other digestive localizations.

c Charlson modified score (excluding malignancies/metastasis).

d Protein energy malnutrition non‐defined according to severity.

e Biological and/or clinical situations potentially source of prescription of artificial nutrition.

f The last 7 or 31 d depending on the endpoint (last 7 or 31 d before death, respectively).

g Cachexia and/or anorexia and/or metabolic disorders and/or mucositis/stomatitis and/or hepatic disorders and/or digestive symptoms and/or respiratory symptoms and/or skin ulceration.

**Table 3:** Factors associated to artificial nutrition use in the last 7 and 31 d before death: multivariate analysis (logistic regressions)

	The last 7 d before death		The last 31 d before death	
	N = 11 723 (2.9%)	*P*	N = 20 429 (5.1%)	*P*
	aOR (95% CI)		aOR (95% CI)	
Sex				
Men (vs women)	1.11 (1.06‐1.15)	**<.001**	1.07 (1.03‐1.10)	**<.001**
Age at death (years)				
≥80 (vs <80)	0.48 (0.45‐0.50)	**<.001**	0.48 (0.46‐0.50)	**<.001**
Year of death				
2014 (vs 2013)	0.99 (0.94‐1.04)	.646	1.01 (0.97‐1.05)	.820
2015 (vs 2013)	0.91 (0.86‐0.96)	**<.001**	0.93 (0.89‐0.97)	**<.001**
2016 (vs 2013)	0.98 (0.93‐1.03)	.410	0.95 (0.91‐0.99)	**.013**
Hospitalization stay duration (days)				
[8‐31] (vs ≤7)	1.54 (1.45‐1.64)	**<.001**	1.84 (1.76‐1.93)	**<.001**
>31 (vs ≤7)	1.44 (1.38‐1.49)	**<.001**	1.73 (1.67‐1.79)	**<.001**
Hospital type				
Speciality (vs non speciality)	1.53 (1.47‐1.59)	**<.001**	1.45 (1.41‐1.50)	**<.001**
Cancer localization				
Digestive^a^ (vs NDHAN^b^)	1.18 (1.14‐1.23)	**<.001**	1.31 (1.27‐1.35)	**<.001**
Head and neck (vs NDHAN^b^)	2.24 (2.09‐2.40)	**<.001**	2.71 (2.57‐2.85)	**<.001**
Metastasis				
Presence (vs no)	0.76 (0.73‐0.79)	**<.001**	0.79 (0.77‐0.82)	**<.001**
Comorbidities (Charlson)^c^				
1 or 2 (vs absence)	1.27 (1.21‐1.32)	**<.001**	1.12 (1.16‐1.24)	**<.001**
3 or 4 (vs absence)	1.26 (1.19‐1.33)	**<.001**	1.19 (1.14‐1.24)	**<.001**
≥5 (vs absence)	1.30 (1.21‐1.39)	**<.001**	1.24 (1.18‐1.31)	**<.001**
Malnutrition				
Mild (vs absence)	2.53 (2.23‐2.88)	**<.001**	2.47 (2.25‐2.71)	**<.001**
Moderate (vs absence)	1.58 (1.49‐1.68)	**<.001**	1.74 (1.67‐1.82)	**<.001**
Severe (vs absence)	1.77 (1.69‐1.85)	**<.001**	2.19 (2.12‐2.27)	**<.001**
Other cases^d^ (vs absence)	1.82 (1.61‐2.05)	**<.001**	1.91 (1.75‐2.09)	**<.001**
Specific biologic/clinical situations^e^				
Presence of at least 1^f^ (vs no)	1.40 (1.34‐1.46)	**<.001**	1.53 (1.47‐1.58)	**<.001**
Dementia				
Presence (vs no)	0.62 (0.54‐0.71)	**<.001**	0.62 (0.56‐0.68)	**<.001**
Chemotherapy use^g^				
Yes (vs no)	1.05 (0.98‐1.11)	.148	1.14 (1.10‐1.18)	**<.001**
Palliative care use^g^				
Yes (vs no)	0.39 (0.37‐0.40)	**<.001**	0.53 (0.51‐0.54)	**<.001**

Note

Bold values: *P*‐values < .05.

Abbreviation: aOR (95% CI), adjusted odd ratio with the 95% confidence interval.

a Digestive cancers: oesophageal, pancreas, and other digestive localizations.

b NDHAN: Non‐digestive and non‐head‐and‐neck cancers.

c Charlson modified score (excluding malignancies/metastasis).

d Protein energy malnutrition non‐defined according to severity.

e Biological and/or clinical situation(s) potentially source of prescription of artificial nutrition.

f Cachexia and/or anorexia and/or metabolic disorders and/or mucositis/stomatitis and/or hepatic disorders and/or digestive symptoms and/or respiratory symptoms and/or skin ulceration.

g The last 7 or 31 d depending on the endpoint (last 7 or 31 d before death, respectively).

### 3.3 Factors associated with artificial nutrition during the last 31 days before death

The factors associated with artificial nutrition during the last 31 days before death were the same factors that were associated with artificial nutrition during the last 7 days before death except for anorexia which was only associated with artificial nutrition during the last 31 days before death. The univariate analysis results are detailed in Table 2 and the multivariate analysis results are detailed in Table 3.

## 4 DISCUSSION

The first important finding of this study was the relatively low proportion of use of artificial nutrition during the last days before death in French cancer patients with a limited expected life‐expectancy: the proportion was 5% in the last month of life and less than 3% in the last 7 days of life. While the French national context is characterized by a high level of medicalization in the last months and weeks of life compared with that of other European countries,^18^ this result indicates that the artificial nutrition management for patients with advanced illness was in accordance with the available clinical guidelines,^10^, ^11^ even though the recommendation was based on sparse evidence‐based medicine findings. Previous studies most often reported higher proportions. For example, four studies^19^, ^20^, ^21^, ^22^ included in the 2011 review^13^ reported frequencies of artificial nutrition in the last week of life from 3% to 50%. However, these four studies were all performed in Asian countries and it is well‐documented that the frequencies of artificial nutrition use may be influenced by cultural/legal differences in end‐of‐life decision making.^23^ Another study,^14^ which used a similar population‐based study design as ours and that was performed on French cancer patients, showed that more than 15% of patients benefited of artificial nutrition during the last month before death, with a slight increase in the last week of life. However, this study reported data from an earlier period (from 2010 to 2013), included patients cared for in rehabilitation units, and focused on patients with advanced upper digestive tract cancers, known to be cancers for which artificial nutrition remains a key component of care.^24^ Artificial nutrition can also induce bronchorrhea, which is a cause of discomfort. This new data has probably reduced the number of prescriptions for artificial nutrition.^25^

The second interesting finding of the study is the factors that were associated with the use of artificial nutrition during the last days of life. Women and, more interestingly, the oldest patients were those who benefited least from artificial nutrition: these two findings were found in previous similar reports.^14^ While we hypothesized that artificial nutrition would most often be used for patients in care‐managed in nonspecialty centers compared to specialty centers (including university hospital and cancer centers), we found the opposite result. It should be expected that the professional caregivers working in specialty centers use less artificial nutrition.^13^, ^22^ Indeed, previous studies reported that professional caregivers working in specialty centers were more concerned about the burden of artificial nutrition in the last weeks of life (and were more reserved about the benefits in terms of the alleviation of symptoms).^20^, ^26^ However, the study by Kempf et al, which used a similar design as was used in our study, showed a comparable result.^14^ One partial explanation may be the deficiency of robust predictive factors of dying. Future research should provide tools or scores to improve this prediction in the last days of life to better manage the end‐of‐life period. Unsurprisingly, the use of artificial nutrition was more frequent in patients with digestive and head‐and‐neck cancers compared to its use in patients with other localizations. At of the end of life, artificial nutrition is often a key component of cancer care for these specific cancers.^27^ In addition, while the use of artificial nutrition is not recommended in patient with short life‐expectancy, the decision to stop or withdraw artificial nutrition may be even harder for both the medical staff and the family. In accordance with the guidelines, the presence of metastasis and increased comorbidities should theoretically be associated with less use of artificial nutrition. This is the case for patients with metastasis, but an increasing number of comorbidities have been associated with a higher use of artificial nutrition. Indeed, patients with comorbidities probably present with a more deteriorated general health status, than patients without comorbidities, which should lead to a decision to stop artificial nutrition. The literature provides discordant results about this association: an increasing number of comorbidities may be associated with a lower^16^ or a higher^14^ use of artificial nutrition. Malnutrition and the presence of medical (biological‐clinical) situations, which are potential indications for artificial nutrition, were all more frequently associated with the presence of artificial nutrition as a compensation for an alteration of general health status. However, for some items, it is well‐known that artificial nutrition was not effective.^11^ Bronchial congestion (as a consequence of lower albuminemia due to hypercatabolism) and nausea/vomiting/diarrhea can be worsened by enteral nutrition. Malnutrition, cachexia, and metabolic disorders, as a consequence of inflammatory phenomena, are generally not improved by artificial nutrition. Despite the growing evidence against artificial nutrition use among patients with advanced dementia, little is known about the perspectives of the healthcare team.^28^ In this large population, we can say that healthcare providers were widely in accordance with the current evidence‐based knowledge about artificial nutrition for patients with dementia at the end of life. One important finding was the association found between a less use of artificial nutrition for patients labeled as being in “palliative care.” In a care model in which the palliative approach is fully integrated into the standard practice, the withdrawal of artificial nutrition is more likely to be understood by cancer patients and their relatives. The role of palliative care specialists is not only to provide care to patients with complex clinical needs but also to assist medical teams with ethically challenging decision making.^29^

Despite these satisfactory findings, it is necessary to question the last remaining obstacles to the reduction in artificial nutrition use in the French context. Since the recent French law (2016), called the French Claeys‐Leonetti Law (LOI n° 2016‐87 du 2 février 2016 créant de nouveaux droits en faveur des malades et des personnes en fin de vie), the notion of “treatment” includes artificial nutrition that allows withdrawing it in limited life expectancy situations. Since artificial nutrition is a treatment, the initiation, termination, and withholding of it must be medically and/or ethically (and consequently legally) justified.^12^ Beyond medical, ethical or legislative factors, the thought processes that influence decision‐making regarding the issues of implementation, maintaining, or withdrawing artificial nutrition^30^ should be explored. Strong opinions surrounding these decisions are rooted in beliefs, representations, and personal and cultural experiences, referring to a basic physiological need associated with psychological, social, and symbolic significance.^30^, ^31^, ^32^ The perspectives of patients/families and professionals provide the basis for decisions regarding artificial nutrition. First, the patients and families may believe that: (a) regarding maintaining/implementing artificial nutrition: it may help them to survive by preventing dehydration and increasing physical strength, it provides a primary necessity^33^, ^34^, ^35^ required by all human beings, it is supported by their religion, and it is a symbol of their families' love^33^; (b) while withdrawing/stopping artificial nutrition may be related to an act leading to death^30^ and, may give the perception that the health care team is negligent.^32^ Second, professionals' beliefs may contribute to the decision about artificial nutrition at the end of life due to the significant influence they have on patients/families in the setting of a trusted caregiver relationship. Physicians who do not frequently participate in the care of terminally ill patients are more likely to consider artificial nutrition as necessary,^31^ to consider the discontinuation as a form of intentional death‐hastening,^36^ and worry about being accused of performing euthanasia if they stop artificial nutrition.^37^ The more‐familiar professionals would argue that artificial nutrition only prolongs the dying process.^12^ Last, the meaning of continuing nutrition at the end‐of‐life differs according to cultural aspects. In western cultures, eating is associated with survival and lack of nutrition is related to death. Elsewhere, nobody dies hungry (Taiwanese belief)^31^ or stops eating to prepare for a dignified death (Hindu tradition). The discrepancies between the different points of view (families/patients vs professionals) should be reduced.^38^ Legal documentation, patients' written wishes, and expressed wishes to a (familial or professional) proxy may prevent disagreements and misunderstandings and may facilitate the final decisions.^32^ Providers must encourage early open conversations with patients/families regarding the course of the disease and the approaching end of life, ensuring the respect of their preference regarding terminal care and the place of death.^39^ The professionals must provide education and guidance about the risks and benefits involved in artificial nutrition and communicate clearly about the limited evidence of its beneficial effects. This communication involves ethical challenges that have been widely debated in the last decades.

### 4.1 Some limitations should be discussed

The generalizability of our results should be discussed. Only patients who died in hospitals were studied, and future studies may explore the phenomenon in other conditions of death, such as patients whose care was managed in rehabilitation centers and at home.

The retrospective design prevented us from truly exploring the relations between the time of the decision to pursue artificial nutrition or not and to implement artificial nutrition or not and the expected prognosis of the patient. Indeed, the 4 last weeks that were considered in the study were probably not the 4 last weeks expected by the physicians at the present time. The trajectory of cancer patients is not easily predictable, even in the last days. Physicians have been found to be overoptimistic regarding the prognosis of terminally ill patients.^40^ The maintenance of artificial nutrition may be due to an overestimation of the prognosis. However, surprisingly, similar results were found between the analysis of the last 7 days and the last 31 days. A prospective design would more appropriate to link the decision to stop/maintain artificial nutrition with the expected time until death.

The accuracy of the findings, due to the source of the data (an administrative registry), depends on the coding rules and the skills of the encoders.^41^ These databases were originally designed for the optimization of funding allocation of the French health facilities. We may hypothesize that the coding procedures might be slightly guided by this first objective, leading to misestimations. The absence of artificial hydration, not catched by the French classification of medical acts, might underestimate the global prevalence of “artificial nutrition and hydration.” Future studies, based on other sources of data, should be used to confirm some assertions.

The expectations of prescribers and patients/families should be investigated as key items to assess the quality and adequacy of artificial nutrition at the end of life. Future studies should further explore these aspects using mixed approaches (quali‐quantitative studies).

## 5 CONCLUSION

This study indicates that the use of artificial nutrition near the end of life is rather low, which is in keeping with current clinical guidelines. The identification of factors associated with the use of artificial nutrition, such as cancer localization, presence of comorbidities or specific symptoms, may help to better manage its use, and may improve communication with patients, families, professionals, and health policy makers.

## CONFLICT OF INTEREST

None declared.

## AUTHOR CONTRIBUTIONS

Karine Baumstarck contributed to conception and design, interpretation of data, drafting and writing of manuscript. Laurent Boyer contributed to conception and design, coordination, methodology guidance, interpretation of data, drafting and writing of manuscript. Vanessa Pauly and Veronica Orleans involved in analysis of data. Anthony Marin contributed to conception and design, analysis of data, interpretation of data. Guillaume Fond involved in methodology guidance and interpretation of data. Lucas Morin contributed to methodology guidance and interpretation of data. Pascal Auquier involved in interpretation of data. Sébastien Salas contributed to conception and design, coordination, interpretation of data, drafting and writing of manuscript. All the authors contributed to revision and approval of final version of the manuscript.

## Data Availability

Data sharing; research data are not shared.
