# Supplementary material for: Use of artificial nutrition near the end of life: Results from a French national population‐based study of hospitalized cancer patients
Source: Cancer Med. 2019 Nov 26;9(2):530–40. doi: 10.1002/cam4.2731 (PMC6970047; doi:10.1002/cam4.2731)
Supplement: Supplementary file 1 [file CAM4-9-530-s001.docx]

**Appendix. ICD-10 and CCAM codes**

- Enteral and parenteral nutrition: HSLF001, HSLD001, HSLF002, HSLF003, HSLD002.
- Malnutrition: mild malnutrition E441; moderate malnutrition E440; severe malnutrition E43; undefined severity E40, E41, E42, E45, E46.
- Cachexia R64.
- Anorexia R630.
- Metabolic disorders: dysnatremia: E8718, E8710, E8700, E8718, E8710; dyskaliemia: E8758, E8768, E8760, E8750; others: E86, E872, E780, E8350, E8358, E785, E880, E833, E8708, E878, E834, E877, E883, E782, E873, E890, E8351, E788, E790, E831, E756, E755, E789, E784, E891, E781, E90, E722, E806, E854, E853, E874, E840, E859, E889, E888, E801, E807, E838, E858, E713, E848, E898, E720, E751, E752, E786, E802, E804, E893, E728, E740, E800, E830, E841, E850.
- Mucositis/stomatitis: K123, K121, K137, K117, K120, K112, K102, K122, K148, K068, K130, K140, K088, K029, K076, K113, K132, K028, K108, K089, K036, K047, K052, K053, K074, K081, K103, K118, K041, K046, K048, K083, K010, K022, K032, K038, K042, K045, K049, K051, K061, K114, K134, K135, K145, K146, K123, K121, B370.
- Hepatic disorders: K720 (acute and subacute hepatic failure), K703 (alcoholic cirrhosis of liver), K729 (hepatic failure), K766 (portal hypertension), K746 (other and unspecified cirrhosis of liver), K767 (hepatorenal syndrome), K710 (toxic liver disease with cholestasis), K750 (abscess of liver), K704 (alcoholic hepatic failure), K768 (other specified diseases of liver), K721 (chronic hepatic failure), K700 (alcoholic fatty liver), K760 (Fatty (change of) liver, not elsewhere classified), K763 infarction of liver), K762 (central hemorrhagic necrosis of liver), K701 (alcoholic hepatitis), K712 (toxic liver disease with acute hepatitis), K761 (chronic passive congestion of liver), K740 (hepatic fibrosis), K716 (toxic liver disease with hepatitis, not elsewhere classified), K765 (hepatic veno-occlusive disease), K711 (toxic liver disease with hepatic necrosis), K717 (toxic liver disease with fibrosis and cirrhosis of liver), K709 (alcoholic liver disease, unspecified), K758 (other specified inflammatory liver diseases), K719 (toxic liver disease, unspecified), K770-8 (liver disorders in diseases classified elsewhere), K744 (secondary biliary cirrhosis), K769 (liver disease, unspecified), K702 (alcoholic fibrosis and sclerosis of liver), K718 (toxic liver disease with other disorders of liver), K743 (primary biliary cirrhosis), K752 (nonspecific reactive hepatitis), K739 (chronic hepatitis, unspecified), K730 (chronic persistent hepatitis, not elsewhere classified), K732 (chronic active hepatitis, not elsewhere classified), K745 (biliary cirrhosis, unspecified), K751 (phlebitis of portal vein), K759 (inflammatory liver disease, unspecified), K715 (toxic liver disease with chronic active hepatitis), K738 (other chronic hepatitis, not elsewhere classified), K742 (hepatic fibrosis with hepatic sclerosis), K754 (autoimmune hepatitis), K764 (peliosis hepatis), K713 (toxic liver disease with chronic persistent hepatitis), K741 (hepatic sclerosis).
- Digestive symptoms: R18 (ascites), R11 (nausea and vomiting), R13 (aphagia and dysphagia), R104 (abdominal pain).
- Respiratory symptoms: R060 (dyspnea), R042 (hemoptysis), R05 (cough), R092 (respiratory arrest), R068 (other abnormalities of breathing), R064 (hyperventilation), R093 (abnormal sputum), R048 (hemorrhage from other sites in respiratory passages), R090 (asphyxia and hypoxemia), R062 (wheezing), R063 (periodic breathing).
- Skin ulcerations: I830 (varicose veins of lower extremities with ulcer), I832 (varicose veins of lower extremities with both ulcer and inflammation), L89 (pressure ulcer), L97 (non-pressure chronic ulcer of lower limb), L984 (non-pressure chronic ulcer of skin).
- Dementia F00, F01, F02, F03, F051, G30, G31.
- Chemotherapy use: Z51.1 or ABLB006, AFLB003, AFLB013, EBLF002, EBLF003, ECLF005, ECLF006, EDLF014, EDLF015, EDLF016, EDLF017, EDLF018, EDLF019, EDLF020, EDLF021, EELF004, EELF005, GGLB001, GGLB008, HPLB002, HPLB003, HPLB007, ZZLF004, ZZLF900.
- Palliative care use: Z515 or ‘inpatient palliative care support bed.
